# Supplementary material for: Modeling of chemo-radiotherapy targeting growing vascular tumors: A continuum-level approach
Source: PLoS One. 2025 Jan 15;20(1):e0301657. doi: 10.1371/journal.pone.0301657 (PMC11734981; doi:10.1371/journal.pone.0301657)
Supplement: S1 File — This file includes: Section I: Boundary conditions; Section II: Parameters for therapy-free model, bevacizumab and docetaxel implementation; Section III: Non-dimensionalized model; Section IV: Oxygen Sensitization parameter determination, Section V: Simulations in structured and unstructured meshes. (PDF) [file pone.0301657.s001.pdf]

Modeling of chemo-radiotherapy targeting growing vascular tumors: a continuum-level approach: Supplementary Material

I. Lampropoulos

School of Chemical Engineering, National Technical University of Athens, Iroon Polytechneiou 9, Zografou 157 80, Athens, Greece

M. Koutsi

Department of Mechanical and Manufacturing Engineering, University of Cyprus, 1 Panepistimiou Avenue 2109 Aglantzia, Nicosia P.O. Box 20537, 1678 Nicosia, Cyprus

M. Kavousanakis

School of Chemical Engineering, National Technical University of Athens, Iroon Polytechneiou 9, Zografou 157 80, Athens, Greece

CONTENTS

I. **Boundary conditions** 3

II. **Parameters for therapy-free model, bevacizumab and docetaxel implementation** 3

III. **Non-dimensionalised model** 5

III.A. Mass balances 6

III.B. Momentum balances 8

III.C. Auxiliary expressions 8

IV. **Oxygen Sensitization parameter determination** 9

V. **Simulations in structured and unstructured meshes** 9

References 10

I. BOUNDARY CONDITIONS

In the first section we present the boundary conditions for our simulations. For a more thorough explanation on the subject, we refer the reader to our previous work[1] and especially the supplementary material accompanying the referred manuscript. All values presented in this section are referring to non-dimensionalised units. The model's non-dimensionalisation is presented in detail in section III.

We summarise boundary conditions in Table S. I. We signify the domain's,Ω, boundary as Γ and the sections of the domain's boundary where -for a fluid phase, i- the expression $\vec{u}_i \cdot \vec{n} < 0$ is true ($\vec{n}$ being the normal vector), are labeled as Γ^{inflow}. Lastly, we denote the pair $(x_{bd}, y_{bd}) = \{(x, y) \in \Gamma\}$.

S. I Boundary conditions for the model's variables.

**Variable solved**	**Boundary condition**	**Boundary section**
θ_i	$\theta_i\,(x_{bd}, y_{bd}, t) = \theta_i(x, y, 0)$	Γ^{inflow}
c	$\nabla c \cdot \vec{n} = 0$	Γ
g	$\nabla g \cdot \vec{n} = 0$	Γ
a	$\nabla a \cdot \vec{n} = 0$	Γ
w	$\nabla w \cdot \vec{n} = 0$	Γ
$\vec{u}_i$	$\boldsymbol{\sigma}_i \cdot \vec{n} = 0$	Γ
$\vec{u}_{int}$	$\vec{u}_{int}\,(x_{bd}, y_{bd}, t) = (0, 0)$	Γ

II. PARAMETERS FOR THERAPY-FREE MODEL, BEVACIZUMAB AND DOCETAXEL IMPLEMENTATION

The parameter values used as a default for the therapy-free model are summarised in Table S. II.

The parameter values used for the inclusion of both cytotoxic chemotherapy and anti-angiogenic therapy are placed in Table S. III.

S. II Dimensionless parameter values for the basic/therapy-free model.

Parameter	Value/expression	Description	Source
$k_{m,c}$	2.0	Cancer cell mitosis rate constant	2
$k_{d,h}$	0.15	Healthy cell death rate constant	2
$k_{d,c}$	0.075	Cancer cell death rate constant	2
c_p	0.25	Threshold nutrient concentration for mitosis	2
c_{c_1}	0.2	Nutrient concentration regulating cellular death rate	2
c_{c_2}	0.1	Threshold nutrient concentration for cellular death rate	2
k_{occ}^{mv}	0.095	Mature vessel occlusion rate constant	3
k_{occ}^{yv}	0.135	Young vessel occlusion rate constant	3
k_{ang}	$0.005/g_{init}$	Angiogenesis rate constant	1
k_{mat}^{ves}	0.025	Capillary maturation rate constant	1
c_α	0.05	Threshold nutrient concentration for angiogenesis rate	2
p_{crit}^{mv}	0.31	Critical pressure for mature vessel occlusion	3
p_{crit}^{yv}	0.26	Critical pressure for young vessel occlusion	3
ε	0.01	Threshold interstitial fluid volume fraction for angiogenesis rate	2
h	0.2	Smoothness parameter for vessel occlusion function	2
D_c	1.0	Nutrient diffusion coefficient	2
$k_{c,h}$	0.01	Consumption rate constant for sustenance of healthy cells	2
$k_{c,c}$	0.01	Consumption rate constant for sustenance of cancer cells	2
$k_{cm,h}$	0.1	Consumption rate constant of nutrient used for mitosis of healthy cells	2
$k_{cm,c}$	$k_{cm,h} \cdot k_{m,c}$	Consumption rate constant of nutrient used for mitosis of cancer cells	2
Λ	0.1	Tension constant	2
μ_i	10.0	Dynamic viscosity	2
$d_{i,j}$	1.0	Drag coefficient	2
k_{g_1}	$4 \cdot g_{init}$	Angiogenesis parameter	1
k_{g_2}	g_{init}	Angiogenesis parameter	1
χ_g	134.6	Chemotaxis parameter	1
ω	3.0	Angiogenesis rate multiplier	1
ν	25.0	Angiogenesis parameter	1
m	1.0	Angiogenesis parameter	1
D_g	0.05	VEGF diffusion coefficient	1
k_{sec}	10^{-5}	VEGF secretion rate constant	1
k_{cang}	$6.57 \cdot 10^{-6}$	VEGF consumption rate constant due to angiogenesis	1
$k_{d,g}$	0.0264	VEGF decay rate constant	1
R_{tissue}	30	Radius of modelled tissue (circular domain, Ω)	-

S. III Dimensionless parameter values used in radiochemotherapy simulations.

**Parameter**	**Value/expression**	**Description**	**Source**
D_a	0.043	Bevacizumab's diffusion coefficient	1
$k_{ap,yv}$	$2.66 \cdot 10^{-5}$	Bevacizumab induced apoptosis rate constant (non-matured capillaries)	1
$k_{cap,yv}$	$3.49 \cdot 10^{-8}$	Bevacizumab's consumption rate constant due to non-matured capillaries' apoptosis	1
ϕ	0.0	Mature vessels resilience parameter	-
$k_{n,g}$	0.3405	VEGF's neutralization's rate constant	1
$k_{n,a}$	0.221	Bevacizumab's consumption due to VEGF neutralisation rate constant	1
a_{50}	1.0	Bevacizumab's median effective dose	1
$k_{rep,a}$	0.0194	Bevacizumab's replenishment rate constant through vasculature	1
$k_{d,a}$	$4.65 \cdot 10^{-5}$	Bevacizumab's decay rate constant	1
$k_{el,a}$	0.097	Bevacizumab's expulsion rate constant	1
T_{bev}	21	Bevacizumab's sessions period	4
a_{inj}	1883.5	Bevacizumab's dose administered per session	1
D_w	0.1	Docetaxel's diffusion coefficient	1
$k_{ap,h}$	60.0	Healthy cell docetaxel induced apoptosis rate constant	3
$k_{ap,c}$	60.0	Cancer cell docetaxel induced apoptosis rate constant	3
$k_{cn,h}$	0.079	Docetaxel's consumption by healthy cells rate constant	1
$k_{cn,c}$	0.079	Docetaxel's consumption by cancer cells rate constant	1
l_{cr}	0.28	Critical proliferation value	3
h_d	$0.01 \cdot h$	Steepness parameter for the kinetic terms of docetaxel induced apoptosis	3
w_m	2.0	Threshold docetaxel's concentration for cellular drug induced apoptosis	3
$k_{rep,w}$	0.42	Docetaxel's replenishment rate constant through vasculature	1
$k_{d,w}$	$1.62 \cdot 10^{-3}$	Docetaxel's decay rate constant	1
$k_{el,w}$	5.62	Docetaxel's expulsion rate constant	1
T_{doc}	21.0	Docetaxel's sessions period	5
w_{ing}	5.0	Docetaxel's dose administered per session	1

III. NON-DIMENSIONALISED MODEL

In this paragraph, we present a non-dimensionalised version of our model, and in particular of the extended version of our model incorporating the effect of administered chemotherapy.

III.A. Mass balances

We perform the following non-dimensionalisation (volume fractions, θ_i, are already dimensionless):

$$t' = k_{m,h} \cdot t, \vec{x}' = \frac{\vec{x}}{L_o}, \vec{u}_i' = \frac{\vec{u}_i}{k_{m,h} L_o}, p_i' = \frac{p_i}{\Lambda}, c' = \frac{c}{c_v}, g' = \frac{g}{g_v}, a' = \frac{a}{a_v}, w' = \frac{w}{w_v}. \tag{III.1}$$

We define L_o equal to the radius of the initial cancer seed in the system.

For the cellular species, the resulting dimensionless mass balance equations read:

$$\frac{\partial \theta_h}{\partial t} + \nabla \cdot (\theta_h \vec{u}_h') = \theta_h \theta_{int} \left(\frac{c'}{c_p^* + c'} \right) - k_{d,h}^* \theta_h \left(\frac{c_{c_1}^* + c'}{c_{c_2}^* + c'} \right) - k_{ap,h}^* \theta_h \frac{w'}{w_m^* + w'} \mathcal{H}(l_h^* - l_{cr}^*, h_d^*), \tag{III.2}$$

$$\begin{aligned} \frac{\partial \theta_c}{\partial t} + \nabla \cdot (\theta_c \vec{u}_c') = {} & k_{m,c}^* \theta_c \theta_{int} \left(\frac{c'}{c_p^* + c'} \right) - k_{d,c}^* \theta_c \left(\frac{c_{c_1}^* + c'}{c_{c_2}^* + c'} \right) \\ & - k_{ap,c}^* \theta_c \frac{w'}{w_m^* + w'} \mathcal{H}(l_c^* - l_{cr}^*, h_d^*) \\ & - k_{rad}^* \theta_c \cdot \sum_{i=1}^{N_{rad}} H\left(t' - t_{rad,i}^*\right) \cdot e^{-r_t^* \left(t' - t_{rad,i}^*\right)}, \end{aligned} \tag{III.3}$$

$$\begin{aligned} \frac{\partial \theta_{yv}}{\partial t} + \nabla \cdot \left(\theta_{yv} \vec{u}_{yv}'\right) + \chi_g^* \nabla \cdot (\theta_{yv} \nabla g') = {} & k_{ang}^* g' (\theta_{yv} + \theta_{mv}) \left(\frac{\theta_{int}}{\epsilon + \theta_{int}} \right) \\ & - k_{occ}^{yv^*} \theta_{yv} \mathcal{H}(\theta_h p_h' + \theta_c p_c' - p_{crit}^{yv^*}, h^*) \\ & - k_{mat}^{ves^*} \frac{k_{g1}^* g'}{(g' + k_{g2}^*)^2} \frac{\theta_{yv}}{(\theta_{yv} + m)^a} - k_{ap,yv}^* a' \theta_{yv}, \end{aligned} \tag{III.4}$$

$$\begin{aligned} \frac{\partial \theta_{mv}}{\partial t} + \nabla \cdot (\theta_{mv} \vec{u}_{mv}') = {} & - k_{occ}^{mv^*} \theta_{mv} \mathcal{H}(\theta_h p_h' + \theta_c p_c' - p_{crit}^{mv^*}, h^*) \\ & + k_{mat}^{ves^*} \frac{k_{g1}^* g'}{(g' + k_{g2}^*)^2} \frac{\theta_{yv}}{(\theta_{yv} + m)^a} \end{aligned} \tag{III.5}$$

$$\theta_{int} = 1 - \theta_h - \theta_c - \theta_{yv} - \theta_{mv}, \tag{III.6}$$

where:

$$k_{m,c}^* = \frac{k_{m,c}}{k_{m,h}}, k_{d,h}^* = \frac{k_{d,h}}{k_{m,h}}, k_{d,c}^* = \frac{k_{d,c}}{k_{m,h}}, k_{ap,h}^* = \frac{k_{ap,h}}{k_{m,h}}, k_{ap,c}^* = \frac{k_{ap,c}}{k_{m,h}}, l_h^* = \frac{l_h}{k_{m,h}}, l_c^* = \frac{l_c}{k_{m,h}},$$
$$l_{cr}^* = \frac{l_{cr}}{k_{m,h}}, h_d^* = \frac{h_d}{k_{m,h}}, w_m^* = \frac{w_m}{w_v}, \chi_g^* = \frac{\chi_g g_v}{k_{m,h} L_o^2}, k_{occ}^{mv^*} = \frac{k_{occ}^{mv}}{k_{m,h}}, k_{occ}^{yv^*} = \frac{k_{occ}^{yv}}{k_{m,h}}, k_{ang}^* = \frac{k_{ang} g_v}{k_{m,h}},$$
$$k_{mat}^{ves^*} = \frac{k_{mat}^{ves}}{k_{m,h}}, k_{g1}^* = \frac{k_{g1}}{g_v}, k_{g2}^* = \frac{k_{g2}}{g_v}, k_{ap,yv}^* = \frac{k_{ap,yv} a_v}{k_{m,h}}, k_{ap,mv}^* = \frac{k_{ap,mv} a_v}{k_{m,h}}, c_p^* = \frac{c_p}{c_v}, c_{c_1}^* = \frac{c_{c_1}}{c_v},$$
$$c_{c_2}^* = \frac{c_{c_2}}{c_v}, h^* = \frac{h}{\Lambda}, p_{crit}^{yv^*} = \frac{p_{crit}^{yv}}{\Lambda}, p_{crit}^{mv^*} = \frac{p_{crit}^{mv}}{\Lambda}. k_{rad}^* = \frac{k_{rad}}{k_{m,h}}, r_t^* = \frac{r_t}{k_{m,h}}, t_{rad}^* = k_{m,h} \cdot t_{rad}.$$

The dimensionless mass balance equations for chemical species are:

$$\begin{aligned} -D_c^* \nabla^2 c' = {} & (\theta_{mv} + \theta_{yv}) (1 - c') \\ & - k_{c,h}^* \theta_h c' + k_{c,c}^* \theta_c c' - \left(k_{cm,h}^* \theta_h + k_{cm,c}^* \theta_c\right) \theta_{int} \left(\frac{c'}{c_p^* + c'}\right), \end{aligned} \tag{III.7}$$

$$-D_g^* \nabla^2 g' = k_{sec}^* (\theta_h + \omega \cdot \theta_c) \frac{c'}{(c' + c_a^*)^2} - k_{cang}^* (\theta_{yv} + \theta_{mv}) g' - k_{d,g}^* g' - k_{n,g}^* \frac{g' a'}{a_{50}^* + a'}, \tag{III.8}$$

$$-D_a^* \nabla^2 a' = k_{rep,a}^* (\theta_{mv} + \theta_{yv})(a_c^* - a') - k_{d,a}^* a' - k_{n,a}^* \frac{g' a'}{a_{50}^* + a'} - k_{cap,yv}^* a' \theta_i, \tag{III.9}$$

$$-D_w^* \nabla^2 w' = k_{rep,w}^* (\theta_{mv} + \theta_{yv})(w_c^* - w') - k_{d,w}^* w' - \sum_i^{h,c} k_{cn,i} \theta_i \mathcal{H}\left(l_i^* - l_{cr}^*, h_d^*\right), \tag{III.10}$$

where

$$a_c^* = \frac{a_c}{a_v} = a_{inj}^* \sum_{i=1}^{N_{bev}} H(t' - t_{inj,i}^{bev^*}) e^{-k_{el,a}^* (t' - t_{inj,i}^{bev^*})},$$

$$w_c^* = \frac{w_c}{w_v} = w_{inj}^* \sum_{i=1}^{N_{doc}} H(t' - t_{inj,i}^{doc^*}) e^{-k_{el,w}^* (t' - t_{inj,i}^{doc^*})},$$

and

$$D_c^* = \frac{D_c}{k_{rep}L_o^2}, k_{c,h}^* = \frac{k_{c,h}}{k_{rep}}, k_{c,c}^* = \frac{k_{c,c}}{k_{rep}}, \quad k_{cm,h}^* = \frac{k_{cm,h}}{k_{rep}c_v}, k_{cm,c}^* = \frac{k_{cm,c}}{k_{rep}c_v},$$

$$D_g^* = \frac{D_g}{k_{rep}L_o^2}, k_{sec}^* = \frac{k_{sec}}{k_{rep}g_v c_v}, k_{cang}^* = \frac{k_{cang}}{k_{rep}}, c_a^* = \frac{c_a}{c_v} k_{d,g}^* = \frac{k_{d,g}}{k_{rep}}, k_{n,g}^* = \frac{k_{n,g}}{k_{rep}}, a_{50}^* = \frac{a_{50}}{a_v},$$

$$D_a^* = \frac{D_a}{k_{rep}L_o^2}, k_{rep,a}^* = \frac{k_{rep,a}}{k_{rep}}, k_{d,a}^* = \frac{k_{d,a}}{k_{rep}}, k_{n,a}^* = \frac{k_{n,a}g_v}{k_{rep}a_v}, k_{cap,yv}^* = \frac{k_{cap,yv}}{k_{rep}},$$

$$D_w^* = \frac{D_w}{k_{rep}L_o^2}, k_{rep,w}^* = \frac{k_{rep,w}}{k_{rep}}, k_{d,w}^* = \frac{k_{d,w}}{k_{rep}}, k_{cn,h}^* = \frac{k_{cn,h}}{k_{rep}w_v}, k_{cn,c}^* = \frac{k_{cn,c}}{k_{rep}w_v},$$

$$k_{el,a}^* = \frac{k_{el,a}}{k_{m,h}}, k_{el,w}^* = \frac{k_{el,w}}{k_{m,h}}, t_{inj,i}^{bev^*} = t_{inj,i}^{bev} \cdot k_{m,h}, t_{inj,i}^{doc^*} = t_{inj,i}^{doc} \cdot k_{m,h}, a_{inj}^* = \frac{a_{inj}}{a_v}, w_{inj}^* = \frac{w_{inj}}{w_v}.$$

III.B. Momentum balances

The non-dimensionalised momentum balance equations read:

$$\sum_{j,j\neq i} d_{i,j}^* \theta_i \theta_j \left(\vec{u}_j' - \vec{u}_i'\right) - \theta_i \nabla \cdot (\Lambda^* p_i' \boldsymbol{I}) + \nabla \cdot \left[\theta_i \cdot \mu_i^* \left(\nabla \vec{u}_i' + (\nabla \vec{u}_i')^T\right)\right] = \vec{0}, \tag{III.11}$$

for $i, j = h, c, yv, mv, int$, with $d_{i,j}^* = \frac{d_{i,j}}{d_{h,c}}, \Lambda^* = \frac{\Lambda}{d_{h,c}k_{m,h}L_o^2}, \mu_i^* = \frac{\mu_i}{d_{h,c}L_o^2}$.

III.C. Auxiliary expressions

There are a few auxiliary algebraic equations that are necessary for the acquisition of unique solutions, starting with the continuity equation (Eq. (6) main text) which has the following dimensionless form:

$$\sum_i \nabla \cdot (\theta_i \vec{u}_i') + \chi_g^* \nabla \cdot (\theta_{yv} \nabla g') = 0, \text{ for } i = h, c, yv, mv, int, \tag{III.12}$$

The constitutive equations for pressures Eq. (7) (Main text) are non-dimensionalised as follows:

$$p_h' = p_c' = p_{int}' + \Sigma'(\theta_h + \theta_c), \tag{III.13}$$

with $\Sigma'(\theta)$ being:

$$\Sigma'(\theta) = \begin{cases} \frac{(\theta-\theta^*)}{(1-\theta)^2}, & \text{if } \theta \geq \theta^* \\ 0, & \text{otherwise.} \end{cases} \tag{III.14}$$

IV. OXYGEN SENSITIZATION PARAMETER DETERMINATION

As presented in the main text (Eq. (31)) the radiation-induced apoptosis kinetic term for cancer cells that takes oxygen radiosensitization into account is:

$$\mathcal{R}_{ox}^{sens} = \left[k_{rad}^{ox}\theta_c \cdot \sum_{i=1}^{N_{rad}} H\left(t - t_{rad,i}\right) \cdot e^{-r_t\left(t-t_{rad,i}\right)}\right] \cdot \left[1 + \xi_{ox} \cdot \tanh\left(\frac{c(x,y,t)}{c\left(x,y,t=0\right)}\right)\right].$$

In order to compare the outcomes of combination therapy with and without taking into account oxygen as a radiosensitizer, we first had to establish that single radiation therapy would produce an equivalent outcome at the end of it's course in both cases. In other words we had to determine k_{rad}^{ox} value with the view to achieve a survival fraction $SF \approx 34\%$ at the end of the therapy's administration.

Like we did in the basic model, through trial and error, we established that for $k_{rad}^{ox} = 0.11$, the new kinetic term produces the desired outcome. As shown in Fig. S. 1, in both cases, the surface coverage is approximately equal, during and shortly after the therapy's end.

V. SIMULATIONS IN STRUCTURED AND UNSTRUCTURED MESHES

In this section, we present a comparison of the computed cancer cell volume fraction distribution between a structured and an unstructured mesh with an approximately equivalent number of degrees of freedom. This comparison is performed using the basic, therapy-free version of the model. In Fig S. 2(a)-(b), we present snapshots of the cancer cell volume fraction at dimensionless time, $t = 200$. The right panel (b) corresponds to the unstructured mesh simulation featuring irregularities in the morphology of the growing tumor. The bottom panel (c) illustrates the averaged radial cancer cell volume fraction for both meshes. Minor discrepancies are observed between these distributions, indicating that the overall tumor growth dynamics remain -coarsely- unaffected by the mesh selection.

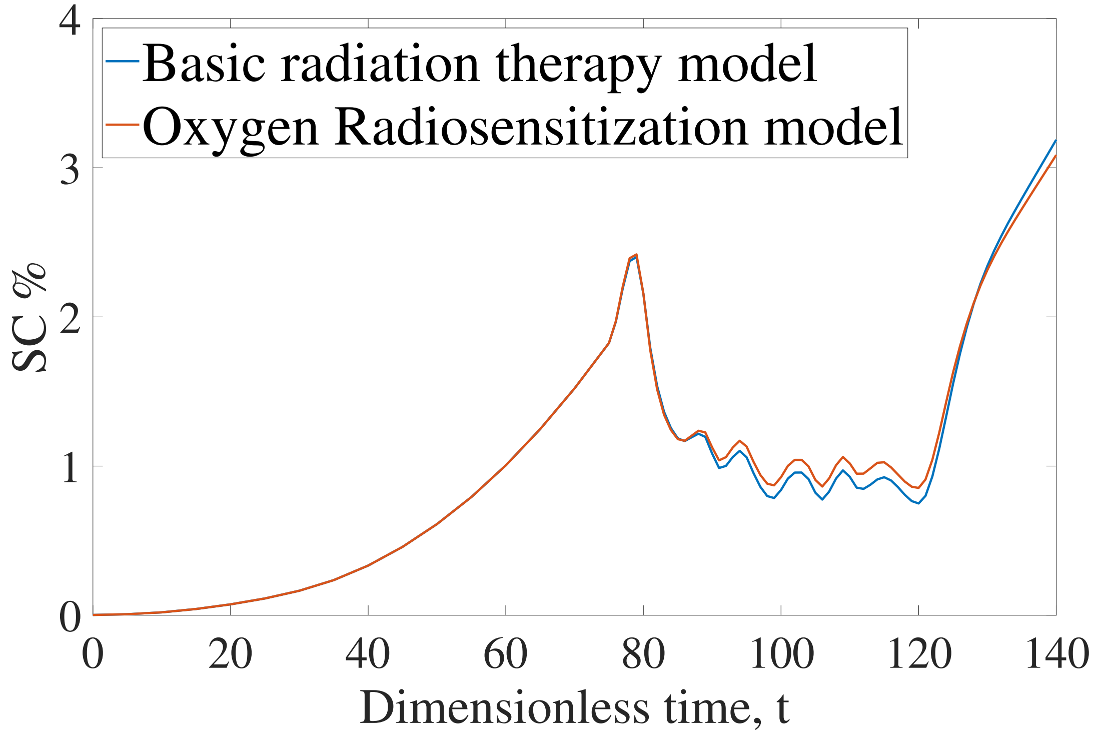

Figure S. 1 Surface coverage for tumor treated with external radiation therapy (blue curve) and the same treatment after taking into account oxygen's cell radiosensitizing property (red curve).

REFERENCES

[1] I. Lampropoulos and M. Kavousanakis, "Application of combination chemotherapy in two dimensional tumor growth model with heterogeneous vasculature," Chemical Engineering Science **280**, 118965 (2023).

[2] M. Hubbard and H. Byrne, "Multiphase modelling of vascular tumour growth in two spatial dimensions," Journal of theoretical biology **316**, 70–89 (2013).

[3] I. Lampropoulos, M. Charoupa, and M. Kavousanakis, "Intra-tumor heterogeneity and its impact on cytotoxic therapy in a two-dimensional vascular tumor growth model," Chemical Engineering Science **259**, 117792 (2022).

[4] A. Wozniak, J. Moon, C. Thomas, K. Kelly, P. Mack, L. Gaspar, D. Raben, T. Fitzgerald, K. Pandya, and D. Gandara, "A pilot trial of cisplatin/etoposide/radiotherapy followed by consolidation docetaxel and the combination of bevacizumab (nsc-704865) in patients with

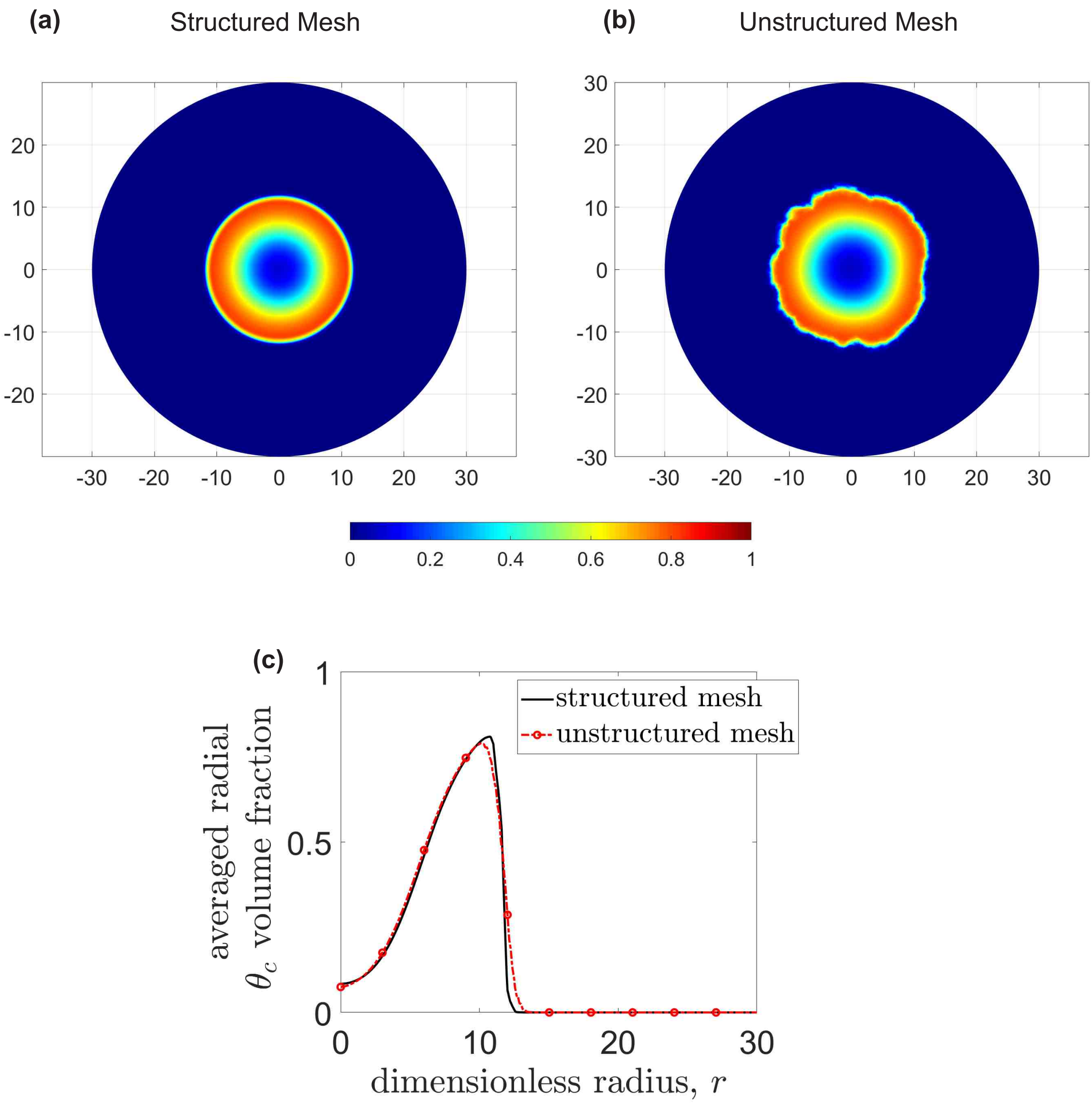

Figure S. 2 Cancer cell volume fraction distribution for a therapy-free simulation at $t = 200$ using (a) an unstructured mesh, and (b) a structured mesh. (c) Averaged radial distribution of cancer cells for structured (black line) and unstructured mesh(dashed-red line with open circles).

inoperable locally advanced stage iii non-small-cell lung cancer: Swog s0533," Clinical Lung Cancer **16**, 340–347 (2015).

[5]H. Kenmotsu and Y. Tanigawara, "Pharmacokinetics, dynamics and toxicity of docetaxel: Why the japanese dose differs from the western dose," Cancer Science **106**, 497–504 (2015), https://onlinelibrary.wiley.com/doi/pdf/10.1111/cas.12647.
